# A Feasibility Trial of Power Up: Smartphone App to Support Patient Activation and Shared Decision Making for Mental Health in Young People

**DOI:** 10.2196/11677

**Published:** 2019-06-04

**Authors:** Julian Edbrooke-Childs, Chloe Edridge, Phoebe Averill, Louise Delane, Chris Hollis, Michael P Craven, Kate Martin, Amy Feltham, Grace Jeremy, Jessica Deighton, Miranda Wolpert

**Affiliations:** 1 Evidence Based Practice Unit University College London and the Anna Freud National Centre for Children and Families London United Kingdom; 2 National Institute of Health Research MindTech MedTech Co-operative Nottingham United Kingdom; 3 National Institute of Health Research Nottingham Biomedical Research Centre Nottingham United Kingdom; 4 Division of Psychiatry and Applied Psychology, Institute of Mental Health, University of Nottingham Nottingham United Kingdom; 5 Bioengineering Research Group, Faculty of Engineering, University of Nottingham Nottingham United Kingdom; 6 Common Room Consulting Limited London United Kingdom

**Keywords:** telemedicine, patient participation, mental health, adolescent

## Abstract

**Background:**

Digital tools have the potential to support patient activation and shared decision making in the face of increasing levels of mental health problems in young people. There is a need for feasibility trials of digital interventions to determine the usage and acceptability of interventions. In addition, there is a need to determine the ability to recruit and retain research participants to plan rigorous effectiveness trials and, therefore, develop evidence-based recommendations for practice.

**Objective:**

This study aimed to determine the feasibility of undertaking a cluster randomized controlled trial to test the effectiveness of a smartphone app, Power Up, co-designed with young people to support patient activation and shared decision making for mental health.

**Methods:**

Overall, 270 young people were screened for participation and 52.5% (142/270) were recruited and completed baseline measures across 8 specialist child mental health services (n=62, mean age 14.66 (SD 1.99) year; 52% [32/62] female) and 2 mainstream secondary schools (n=80; mean age 16.88 [SD 0.68] years; 46% [37/80] female). Young people received Power Up in addition to management as usual or received management as usual only. Posttrial interviews were conducted with 11 young people from the intervention arms (specialist services n=6; schools n=5).

**Results:**

Usage data showed that there were an estimated 50 (out of 64) users of Power Up in the intervention arms. Findings from the interviews indicated that young people found Power Up to be acceptable. Young people reported (1) their motivation for use of Power Up, (2) the impact of use, and (3) barriers to use. Out of the 142 recruited participants, 45.0% (64/142) completed follow-up measures, and the approaches to increase retention agreed by the steering group are discussed.

**Conclusions:**

The findings of this study indicate that the app is acceptable, and it is feasible to examine the effectiveness of Power Up in a prospective cluster randomized controlled trial.

**Trial Registration:**

ClinicalTrials.gov NCT02552797; https://clinicaltrials.gov/ct2/show/NCT02552797 (Archived by WebCite at http://www.webcitation.org/6td6MINP0)

## Introduction

### Background

A minority of the population (17%) will not experience mental health problems in their lifetime [1]. On the basis of the last prevalence study, 10% of children in the United Kingdom have a clinically diagnosable mental health problem, with one of the most prevalent being emotional problems including anxiety and depression, of whom 25% receive support from specialist mental health services [2]. Recent evidence suggests that levels of mental health difficulties in young people are increasing with, for example, 1 in 4 young women experiencing emotional problems [3].

Around 25% to 40% of the population have little knowledge, skills, and confidence to manage their own health and health care (referred to as patient activation) [4]. Empowering patients to be actively involved in the management of their health care and involving them in shared decision making are emphasized in the Health and Social Care Act 2012 [5]. Evidence suggests this may have a range of benefits to health and care [6]; for example, a systematic review found that patients were more likely to adhere to treatment when it was in line with their preferences [7,8]. However, empowering patients to actively manage their health care is not widely practiced [9] and clinicians report being unclear about how to facilitate it [10]. A systematic review of observer-rated studies showed that clinicians rarely facilitate patient involvement and adjust care to patient preferences even less often [11].

Young people want to be actively involved in making decisions about their health care and report feeling more in control of their care when they are included in decisions [12]. Parents also feel that their children should be involved in decisions about their care as it may increase their self-esteem and improve their overall welfare [12]. Furthermore, it is recognized under the United Nations Convention on the Rights of the Child that young people should be involved in all matters that affect them.

Interventions in child mental health settings which include empowering patients to be actively involved have been shown to improve quality of life and satisfaction [13,14], and child and parent experiences of shared decision making have been shown to be associated with higher levels of symptom improvement [15]. Evidence of promise has also emerged from an evaluation of tools supporting young people’s mental health through preparing for discussions, mood tracking, and self-management [16]. Evidence from adult settings suggests that interventions targeting empowerment and patient activation may promote engagement with services and interventions [17]. Nonattendance of appointments in child mental health services is an estimated 15% to 28% [18-23]. Noncollaborative decision making is a key predictor of nonattendance [24]. Introducing resources that promote better accessibility to and integration of care, and that which correspondingly make clinicians’ time more efficient, would be invaluable to guarantee that services such as specialist child mental health services can continue to provide good quality service to as many people as possible.

Despite these benefits, young people are presented with a lack of opportunities for reflection and involvement in the decisions that affect them and can often feel unskilled or unsupported in these situations. This is a barrier to their involvement. Correspondingly, there is a need for the development of appealing and acceptable patient activation and shared decision-making tools that support young people to ask questions independently and raise the issues they want to discuss.

Young people have advised that technology that is engaging, easy to access, informative, empowering, and provides support between sessions would be a particularly useful addition to therapy [25]. The use of technology in some areas of mental health care is recommended by the National Institute for Health and Care Excellence 2011 best practice guidance [26]. Indeed, young people report already using technology as an informal complement to treatment [25]. In Great Britain, 82% of adults use the internet daily and 70% of adults use the internet on smartphones [27]. Some 83% of young people aged 11 to 18 years own a smartphone and use mobile internet daily [28,29]. The growth in the smartphone and tablet market and the high levels of engagement within mobile app usage mean that there has been a rise in the adoption of mobile health care apps. The mobile health care market was estimated to be worth US $25.39 billion in 2017 [30].

Young people, carers, and clinicians report feeling positive about integrating the use of certain apps into interventions for young people in mental health settings [16]. However, the content of many youth mental health apps is not grounded in psychological theory or evidence-based practice [31]. There is a need for evidence from rigorous trials as to the effectiveness of digital interventions for mental health in young people [32], in addition to research investigating how best to integrate these apps into support provision [25]. To plan rigorous trials to examine effectiveness, feasibility trials are needed [33]. In particular, there is a need for feasibility trials to determine (1) the usage and acceptability of digital interventions for youth mental health and (2) the ability to recruit and retain research participants [34,35].

### Aims and Objectives

The aim of this study was to determine the feasibility of examining the effectiveness of a smartphone and tablet app, Power Up, in a prospective cluster randomized controlled trial and to determine the usage and acceptability of Power Up. In addition, we examined the ability to recruit and retain research participants. The app was designed to increase a young person’s patient activation related to their mental health by providing tools to support their voice in therapy, facilitate a more patient-centered approach, and increase shared decision making. The app was developed in partnership with young people and advocates to increase its acceptability to young people. Power Up enables young people to record their questions, plans, decisions, and diary entries and supports young people to identify individuals in their support network with whom they would like to share these entries. By providing a digital space for young people to prepare what they want to bring to conversations about their mental health and well-being, Power Up was designed to empower young people to take an active role in decisions that impact their health and care. Both professionals and young people with lived experience were involved in the design of Power Up, ensuring that the views of all relevant groups were heard during app development.

The objective of the present feasibility trial was to collect the necessary parameters to plan a cluster control effectiveness trial of Power Up. In line with guidelines on conducting feasibility trials [36], the Trial Steering Committee developed Go and No Go criteria to determine whether or not the findings from this study indicated that it would be feasible to examine the effectiveness of Power Up in a full trial.

## Methods

### Overview

A protocol for the feasibility trial was published [37] and registered with trials registries. To determine the feasibility of examining the effectiveness of Power Up in a prospective cluster randomized controlled trial, the Trial Steering Committee agreed the following Go and No Go criteria for the study. In line with guidance on conducting feasibility trials [36], we did not set any criteria related to effectiveness as the aims of this study were to collect the parameters necessary to plan the full trial and ensure an analysis of effectiveness was adequately powered. The criteria for the feasibility study are presented in Textbox 1.

**Go and No Go criteria for the feasibility study.**

Scoring key

- A total score of 0 represents all criteria were fully met, and the study is rated as green meaning the full trial can immediately proceed.
- A total score of 1 to 5 represents some criteria were partially met, and the study is rated as amber meaning the full trial can proceed once relevant criteria have been reviewed and a plan to increase adherence has been agreed by the Trial Steering Committee.
- If 1 criterion is not met (ie, rated as red), the trial is rated as red. The full trial can only proceed if (1) the total score is less than 8 meaning not more than 2 criteria can be rated red and (2) a plan to increase adherence to any red criteria has been agreed by the Trial Steering Committee.

Criteria

1. Ability to recruit and retain sites

- 70% to 100% of sites recruited and retained (green)
- 50% to 69% of sites recruited and retained (amber)
- 0% to 49% of sites recruited and retained (red)

2. Rates of sign up to study for young people approached

- 60% to 100% of young people signed up to participate (green)
- 40% to 59% of young people signed up to participate (amber)
- 0% to 39% of young people signed up to participate (red)

3. Rates of download and usage of Power Up

- 60% to 100% of young people download and use Power Up (green)
- 40% to 59% of young people download and use Power Up (amber)
- 0% to 39% of young people download and use Power Up (red)

4. Young people report Power Up as acceptable in interviews

- 70% to 100% young people report that Power Up is acceptable (green)
- 50% to 69% of young people report that Power Up is acceptable (amber)
- 0% to 49% of young people report that Power Up is acceptable (red)

5. Completion of study measures at baseline and follow-up

- 50% to 100% of young people and carers complete study measures at both baseline and follow-up (green)
- 20% to 49% of young people and carers complete study measures at both baseline and follow-up (amber)
- 0% to 19% of young people and carers complete study measures at both baseline and follow-up (red)

### Changes to Protocol

We had initially planned to only conduct the feasibility trial in specialist Child and Adolescent Mental Health Services (CAMHS). We added a schools strand to the feasibility trial for 2 reasons. First, it became clear from feedback from service users, professionals, and researchers that Power Up was applicable to settings beyond specialist mental health services to empower young people to self-manage emotional well-being. Second, the rate of recruitment from specialist services was slower than anticipated, because of various reasons such as clinician workload, turnover, and either young people not attending appointments, not meeting study inclusion criteria, or both. Correspondingly, the target audience for Power Up expanded during the course of the study from just young people experiencing mental health problems to young people in schools to support self-management of emotional well-being.

### Recruitment

In the feasibility trial, young people’s experiences while using Power Up were compared with young people’s experiences without using the app. Overall, 270 young people were screened for participation across 8 specialist services (n=79) and 2 mainstream secondary schools (n=191) (the demographics are reported in the Results section).

For the specialist services strand of the trial, a wait list control design was employed. Initially, 33 young people were recruited to the control phase of the trial where they received management as usual (average cluster size 4.13 (SD 3.48)). Subsequently, 30 different young people were recruited to the intervention phase of the trial where they were given Power Up to use alongside management as usual (average cluster size 3.75 (SD 3.20)). This study was given a favorable opinion by the Health Research Authority Research Ethics Committee (reference number: 192592). Clinicians identified young people who were aged 11 to 19 years and were in their initial assessment sessions for recruitment to the trial. Once consent had been given, young people completed a questionnaire containing a battery of measures. Study measures included (1) the Patient Activation Measure [4] to assess young people’s empowerment and self-management of their mental health and well-being, (2) the CollaboRATE [38] and the Shared Decision Making Questionnaire 9 [39] to assess shared decision making, (3) the Youth Empowerment Scale—Mental Health [40] to assess young people’s confidence to manage their mental health (ie, self-subscale) and the support they receive from services (ie, service subscale), (4) the Strengths and Difficulties Questionnaire [41] to assess young people’s mental health, and (5) the Experience of Service Questionnaire [42] to assess young people’s experiences within mental health services. In the intervention condition, young people were then provided instructions on how to download and use the app. After 3 months, all participants and clinicians were recontacted by the researchers and asked to complete the same questionnaires. Participants in the intervention phase were also asked if they would like to participate in a short semistructured interview about the acceptability of Power Up.

For the schools strand of the trial, a cluster randomized design was employed. Students in 12 clusters (classes) across 2 schools were randomized at the class level to either receive the app or not. This study was given a favorable opinion by University College London Research Ethics Committee (reference number: 6087/006). Randomization was achieved by using random number generation resulting in 6 intervention arm clusters, with 44 students allocated to receive Power Up (average cluster size 6.50 (SD 1.87)). The remaining 6 clusters were randomized to the control arm, with 50 students allocated to receive management as usual (average cluster size 7.50 (SD 4.04)). Researchers explained the nature of the research to the students before inviting them to take part. Participants gave their written consent and completed a questionnaire containing a similar battery of measures compared with that of the specialist services group, but excluding the shared decision-making measures: (1) the Patient Activation Measure [4] to assess young people’s empowerment and self-management of their mental health and well-being, (2) the Strengths and Difficulties Questionnaire [41] to assess young people’s mental health, (3) the Short Warwick-Edinburgh Mental Well-Being Scale [43] to assess young people’s well-being, and (4) the child-friendly version of the EuroQol five dimension quality of life measure [44] to assess young people’s quality of life and to inform the feasibility of conducting health economic analysis. Subsequently, those randomized to the intervention arm were given verbal instructions on how to use and download the Power Up app. After 6 weeks, all participants were contacted by researchers and asked to complete the same questionnaire measures. Participants in the intervention arm were also invited to participate in a short semistructured interview about the acceptability of Power Up.

### Intervention

**Figure 1:**
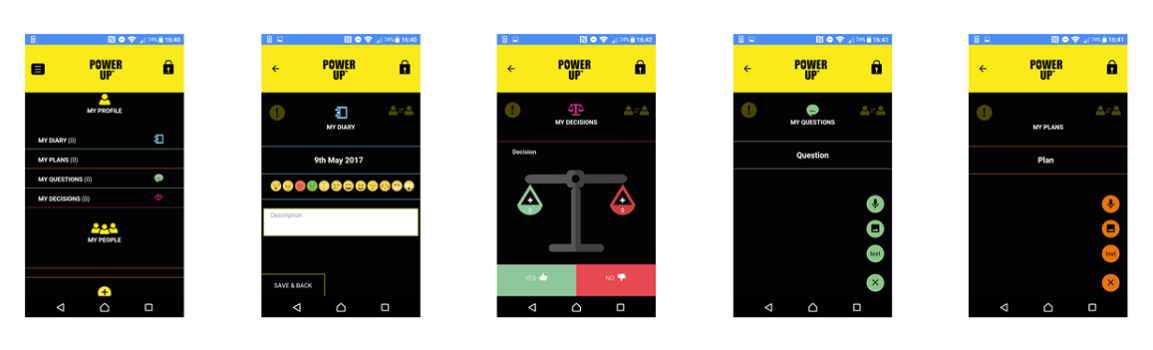
Power Up screenshots.

Power Up was developed based on the theory of patient activation [4] and a scoping review of tools to support young people to be actively involved in decisions about their care [45]. Power Up was designed to be a transdiagnostic and transtherapeutic intervention. To ensure it was accessible to young people, Power Up was co-designed with young people, carers, and clinicians through patient and public involvement workshops and interviews (see [46] for full details on the development of Power Up). A key topic of the codesign sessions was to ensure Power Up was simple and easy to use requiring minimal cognitive load so it could be used by a range of young people with different language skills, literacy levels, and experience of current distress. A mixture of text and icons is used and users can customize the iconography as straight-lined (aimed at older age ranges) or cartoon-style (aimed at younger age ranges). The main features of Power Up are described in Textbox 2 and Figure 1.

**Main features of Power Up.**

- My People: At the center of Power Up, young people can add people in their support network. Users can flag information entered in other sections of the app to specific people in their support network, and all content flagged for sharing with a specific person from other areas of the app is displayed in My People. If an entry is not flagged to My People, it is stored chronologically; otherwise, a young person can prioritize which entries should appear first.
- My Diary: A space for users to express what is going on for them in their daily lives.
- My Plans: A section devoted to adding all plans and goals, including what to do in specific circumstances related to users’ mental health, such as anxiety-provoking situations.
- My Questions: Young people can enter any questions they have or wish to discuss with carers, friends, teachers, or clinicians and keep a record of the answer after it has been discussed.
- My Decisions: A space for young people to work through decisions, weighing up the pros and cons associated with decisions using a visual weighted scale.
- There is the option for all entries in the above sections to be inputted in the form of photo, video, audio, or text.
- Entries such as photos or phone numbers can only be viewed within Power Up, not in the phone’s main library or phonebook.
- Help and Support: A selection of resources that give the young person a series of links to websites and phone numbers. There are a set of prestored resources, however, the young person can also add his or her own. These can be called or visited directly from the app.
- Power Up is secure and password protected.

### Statistical Analysis

To inform the planning of a prospective cluster randomized controlled trial, participant recruitment and retention was captured using the Consolidated Standards of Reporting Trials guidelines. Descriptive statistics were analyzed using SPSS (IBM Corp) [47]. Posttrial interviews were analyzed using thematic analysis [48] in NVivo (QSR International Pty Ltd.) [49].

## Results

### Recruitment and Retention

**Figure 2:**
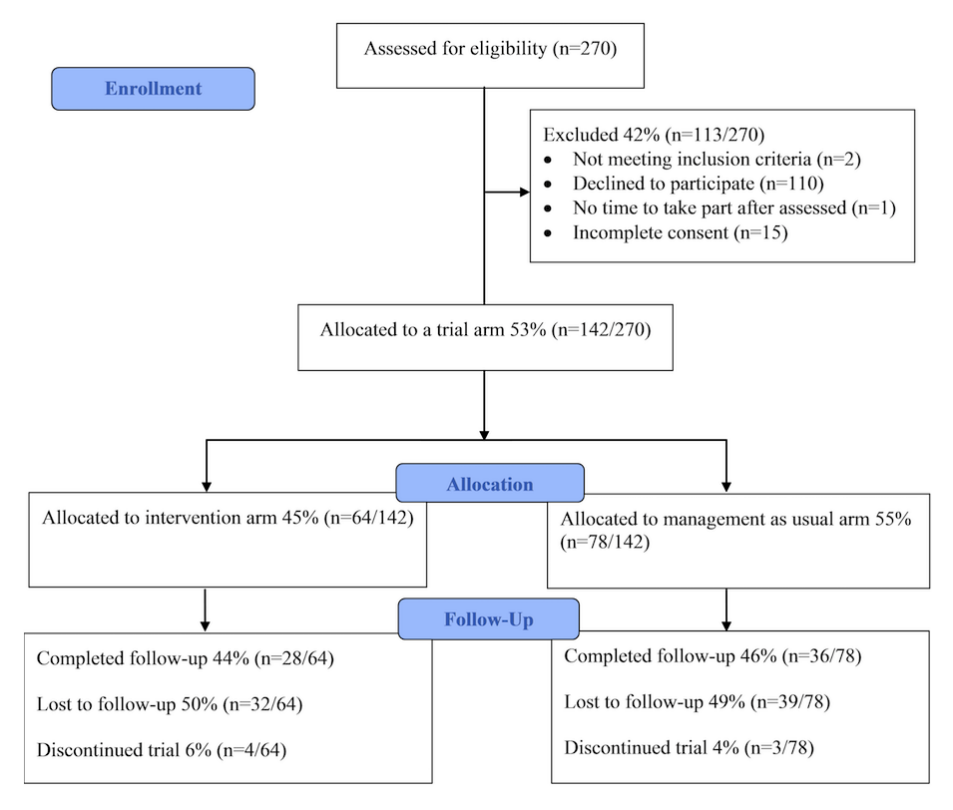
Consolidated Standards of Reporting Trials diagram: combined flow of participants through the study across both specialist services and schools strands of the trial.

Recruitment and retention are reported in the Consolidated Standards of Reporting Trials diagram in Figure 2. During recruitment, 270 young people were assessed for eligibility. A number of young people were screened for participation but not recruited, because of reasons such as refusal and practical barriers such as not having enough time on the day of the research team’s visit; for example, some young people and carers in specialist services were interested in the study but not able to stay to discuss the study as their parking was due to expire after their appointment. A further small proportion was excluded from the trial because of failing to complete all fields on their informed consent forms or failing to return study materials. In total, 142 participants were recruited and completed Time 1 measures (specialist services: n=62, mean age 14.66 (SD 1.99) years, 52% (32/62) female, 42% (26/62) white or white British; schools: n=80; mean 16.88 (SD 0.68), 46% (37/80) female, 26% (21/80) white or white British; see Table 1 for full demographic details). Of those who completed Time 1 measures, 64 (45%, 64/142) adhered to the study protocol and completed Time 2 follow-up assessments. All specialist services and school sites were retained in the study (N=10).

**Table 1:** Participant demographics.

Participant demographics		Child mental health services (n=62)	Schools (n=80)
**Age (years)**			
	Mean (SD)	14.66 (1.99)	16.88 (0.68)
	Range	11-18	16-18
**Gender, n (%)**			
	Female	32 (52)	37 (46)
	Male	26 (42)	40 (50)
	Other	2 (3)	3 (4)
	Prefer not to disclose	2 (3)	—^a^
**Ethnicity, n (%)**			
	White	26 (42)	21 (26)
	Black	7 (11)	14 (18)
	Asian	15 (24)	27 (34)
	Mixed	12 (19)	5 (6)
	Other	1 (2)	12 (15)
	Not reported	1 (2)	1 (1)
**English as first language, n (%)**			
	Yes	52 (84)	52 (65)
	No	7 (11)	26 (33)
	Prefer not to disclose	—	1 (1)
	Not reported	3 (5)	1 (1)
**Registered as disabled, n (%)**			
	Yes	3 (5)	1 (1)
	No	59 (95)	77 (96)
	Prefer not to disclose	—	2 (3)

^a^No participants selected this response.

### User Statistics

We could only determine a number of new users during the entire project time frame from January 2017 to February 2018. The number of active sessions and duration of sessions were available between November 2017 and February 2018 as the app developers upgraded the activity data capture system during the feasibility trial. App usage data are anonymous to comply with data protection and research ethics approvals. Overall, there were 70 new users between January 2017 and February 2018, of which we estimate 20 were nonintervention arm participants (ie, 5 members of the research team, 2 app developers, and 13 clinicians) resulting in an estimated 50 users of Power Up in the intervention arm, out of 64 participants allocated to this arm. There were 13 active users between November 2017 and February 2018 and these users used the app in 89 active usage sessions (corresponding to 6.8 sessions per active user) with an average of 8 min per session.

### Posttrial Interviews

Posttrial interviews were conducted with 11 young people from the intervention arms of the feasibility trial (specialist services n=6; schools n=5; mean age 15.55 (1.86), range=11-17 years). Interviews were audio recorded and transcribed verbatim. Young people described their experiences of using Power Up and of its impact on self-management and well-being, including the context of app use and suggested amendments to the app. The analysis also provided understanding as to barriers faced by young people to downloading and using the app. Finally, interviews indicated the acceptability of Power Up, which will inform the prospective cluster control trial. Findings from the interviews are reported below relating to (1) motivation for use of Power Up, (2) impact of use, and (3) barriers to use (the themes, descriptions, and quotes from the posttrial interviews are fully reported in Textbox 3). We aimed to recruit 10 to 12 young people as we expected this would be sufficient for saturation, which was achieved. We report on these interviews in this study and discuss the views of clinicians, parents and carers on the development of Power Up elsewhere [46] and we have reported in detail on teachers’ views and experiences of the implementation of another digital intervention [50].

**Themes, descriptions, and quotes from posttrial interviews.**

1. Motivation for use
  - Young people outlined different motivations for using Power Up, pertaining to the unique qualities of the app
    - “...I still did find myself using it, especially for the different aspects, like, how you can put key phone numbers on there, as well as, um, as I mentioned before, how you can weigh up decisions and so on, those, those are good aspects, which you wouldn’t be able to do elsewhere…” [Young Person, aged 17 years]; “I used the Diary the most, like; I have quite a few, because I felt it easier to get my feelings down...” [Young Person, aged 14 years]; “I think that if you, like – the way that the Decisions work and the Plans work, it’s really – it’s good because it weighs it up for you.” [Young Person, aged 14 years]; “Um, just if you have, like, any thoughts, so I got bad test results, so I, kind of, logged that, just so, like, I can, like, put it away and then reflect on it when I needed to.” [Young Person, aged 17 years]; “During the holidays, I used the My Plans bit, so what I’m going to do. What I, like, aspirations that I want to do over the holiday.” [Young Person, aged 16 years]
  - The ability to make entries into the app via multiple modalities was evaluated positively by young people
    - “The decision making part was a key factor as well as diary entries, because I’m not a fan of writing, really, so typing it actually was a nice change for once.” [Young Person, aged 17 years]; “I like the video function, definitely, because writing in a diary or something, you actually have to get a pen out and it takes time.” [Young Person, aged 17 years]
  - Young people expressed a need to be able to trust that the technology was a private platform, which could not be accessed by others, unless users chose to share it with them. Power Up represented an essential, secure space for users, allowing young people to feel comfortable to make entries, which were potentially personal and sensitive in nature
    - “Um, I liked that it was locked, because my parents have a tendency to go on my phone.” [Young Person, aged 16 years]; “I guess the best word to describe it would be, I think, safe, because you can say honestly, you can say honestly anything you want, things you wouldn’t share with other people, plans that you want to make for the better that you don’t feel like telling everyone or your parents and it’s a bit of, like, some alone time because some people work best when they’re by themselves.” [Young Person, aged 17 years]; “I like the fact that the PIN, because I feel that’s really, really important because for young people, some aren’t really open about how they feel and how…for someone that is worried the fact that they have a PIN on there is that no one can get in…If they did want to keep it secret, it’s really discreet, that’s what I like.” [Young Person, aged 16 years]; “I think it’s quite, it was, it was very discreet so when using it I didn’t feel like, I didn’t feel really weird...because there’s a, there’s a PIN, no one can really get into it, so I won’t be worried about anyone seeing anything.” [Young Person, aged 16 years]
  - Beyond the inbuilt properties of the digital tool, the interviews elucidated several reasons for young people choosing to use Power Up. The app aided young people to remember important things to either share in a consultation with a health care professional, or to reflect on their thoughts over time
    - “I really loved the app…it’s been so helpful with my appointments because I tend to have big gaps between my appointments… Of three to four weeks, and so I can hardly remember anything and so it was really easy for me to just pick up on little things and then, when I actually get to my appointment, I can just go in, like, chronological order and it was just amazing to be able to do that so I loved it.” [Young Person, aged 16 years]; “Ah, I think the diary bit is good because when you’re—when you’re in that state of mind, you—my mind tends to run away with itself so it’s good to look back on what I’ve thought about and what I’ve said.” [Young person, aged 14 years]; “...because when I write the plans, um, I can write here and I can make sure I do it, because I mostly work in the plans. And in the diary I can look through back and see all the, all the stuff that happened before.” [Young Person, aged 14 years]
  - Young people also sought out Power Up when they could not speak to anyone else about their concerns and emotional experiences. Respondents reported using the app to express their feelings when they were alone
    - “Uh, well my first year score was quite bad, so when I came back home I, my parents were out, they weren’t at home, so I just, like, used the app, like, [inaudible] I thought, like, a good way to get everything out, like immediately...And it just, it helped when I was frustrated and I didn’t like, I felt there was no one to listen to me, but the phone will, so I just threw everything on there.” [Young Person, aged 14 years]; “I think it normally be the later at night because as, you know, night can sometimes be a lonely time for most people…it’s better than just bottling your thoughts up, you can actually, you’ll have your phone, you can go on it and no one needs to know about it, you can just put anything down in the confidential app and it’s just good to have that, especially later at night.” [Young Person, aged 17 years]; “I don’t have anyone that I can talk to, so writing it down, sort of, gives me a bit of relief, so I can just, like, release everything that I’m feeling and something that is written down, I can—it’s there and I can forget about it until I actually have to talk about it. So that was really good.” [Young Person, aged 16 years]
  - Finally, the accessibility of Power Up in the moment was a key motivating factor for young people’s engagement with the technology. Interviewees indicated that the portability of Power Up as an app on their mobile device was fundamental, meaning that they could access it immediately when required in a given situation
    - “I did also do a couple of ones for, like, plans how to deal with, like, situations… I’m very emetophobic, like as soon as I start feeling sick, like, yeah, that’s it, so I did a couple of ones, like how I’m going to deal with it if it happens in a theatre, like how am I going to deal it with it happens in a restaurant, so I did a couple of those I think.” [Young Person, aged 16 years]; “It’s just good to have somewhere that’s stored, like, if it was in a book, for example, you could, you wouldn’t carry a book everywhere you go but your phone is with you roughly 24/7, so whenever you have anything it’s all in this unit or place and it works very nicely.” [Young Person, aged 17 years]; “Um, just to like put, so, like, if I had something that I was worried about, I’d, like, just talk myself through it, so then, I like, on the train home I can just go over what I’d do if that situation happened.” [Young Person, aged 16 years]
2. Impact of use
  - The analysis also illuminated the impact of using Power Up, from allowing young people to derive new insights from documenting their experiences, through to encouraging conversation with others in their support network. Young people explained how using the app had allowed them to see changes in their emotional state over time, marking progression in their psychological journey
    - “I could write it down and then I could look back and see how things have changed over, like, the week or the month and I could see, I went from a really bad phase to a really good phase and it was just, like, good to see progress through that, so that was great.” [Young Person, aged 16 years]; “I’ve had a bit of a transition over these past few weeks, just mentally and everything regarding where I am at CAMHS. And so, so because also the fact that I have a replacement phone now, so after I get in contact with the new e-mail, I’ll re-download it and I’ll start actually afresh. Because then, almost like leaving my old self in the past, so hopefully I’ll start with positive experiences and so on...” [Young Person, aged 17 years]; “I’ve gone through it on my own some nights when I just sit and go through and recall the things that have happened, and so, um, that’s more of just me reviewing how much progress I’ve made and so it was really helpful in terms of that.” [Young Person, aged 16 years]
  - Participating young people identified that a further consequence of using the app was that it had helped them to gain greater understanding about themselves, as well as to clarify their thinking by weighing up the pros and cons of significant issues in a balanced, considered way
    - “...especially with Decisions and so on and the Diary entries because that, because they were there and it’s in an easily accessible place, people do tend to go, go back on their thoughts a lot and because they have been written down it was very easily accessible to go back, change them and actually add them, so to, to, oh yes, get a deeper understanding of yourself.” [Young Person, aged 17 years]; “Um, only when I was on My Diary and I was, like, looking through it and I had a few, like, you know, you can put little emojis, there was a few, like, sad ones, because I felt, like, I was using it more for when I was sad than, like, writing, oh I’m happy today. So I guess that was just more for myself, but then, like, I realised I was just, kind of, using it more for when I’m feeling emotional.” [Young Person, aged 16 years]
  - Young people highlighted that Power Up mediated communication with important people in their support network, facilitating conversation and helping them to share things with others, which they might not have otherwise
    - “I like that I can write everything, um, for myself and my mum can log into my account and check as well… (Young Person’s parent: ‘It’s fine, I mean, there’s things that he hasn’t told me and then I’ve seen them, but he’s writing them in there...’) ...but I was happy that my mum can see all my diary entries and she can only see it.” [Young Person, aged 11 years]; “I do have some, I do have someone in mind who I would mention it to, family wise, because they are going through a similar thing to me and they do, they are struck by boredom a lot, they have nowhere to really share their thoughts. And I’m the person they, kind of, go to, so I’m saying if I’m not always there, because I can’t always be there for them, then there’s this app. So if they want to, like, sit down and talk to me then that will always work and then we can actually share our thoughts on the apps what we have got and see how we are progressing, and get through it together.” [Young Person, aged 17 years]; “I can allocate things to the, like, diary entries to the specific people and so afterwards, because I have, like, 54 diary entries, and so when I, if it got to a point where I want to speak to my therapist, I just click on, like, her name specifically, and all of the diary entries are allocated to her came up specifically.” [Young Person, aged 16 years]
3. Barriers to use
  - Despite young people describing largely positive experiences of Power Up, interviews indicated key barriers impacting engagement with the digital tool. Young people reported that a number of technological difficulties associated with the app occurred throughout the course of the feasibility trial
    - “So if you, so if you go to the Diary section and you stay in one journal without saving and you were just continuously writing thoughts as they flaring up, after five minutes, due to a safety feature implemented it would log you out and then that means that it wouldn’t, um, there was no auto save or backup unfortunately, which means I would have to restart it.” [Young Person, aged 17 years]; “It took a little bit longer to download than another app but I assume that wouldn’t be, that wouldn’t, kind of, affect it in its final form because you wouldn’t need to download the test flight thing and remember code.” [Young Person, aged 16 years]; “Sometimes it, well firstly it would take quite a long time to load the app. And it would, like it would, like, glitch with, like, colours of black and yellow and then it would go blue and then it comes up after that.” [Young Person, aged 14 years]
  - Barriers pertaining to context of app use were also raised. Young people reported disengaging from the app, because of experiencing difficulties in their personal circumstance
    - “Um, so I think I used it about, for like maybe the first month I just did, like, once every week. And afterwards, I think for me life got stressful and then I stopped using it...” [Young Person, aged 17 years]
  - Additionally, it became clear that Power Up had not been fully embedded into mental health services and was seldom incorporated into clinical sessions between young people and their therapists, contrary to expectations
    - “I’m still being introduced into the whole process and I’ve only had two sessions, which would have been an entry session and a session regarding my medication. So possibly in future ones I’ll ask for the integration of it, but I don’t think there, there is any integration at the moment for some people who take CAMHS appointments and so on.” [Young Person, aged 17 years]

## Discussion

### Principal Findings

The aim of this study was to determine the feasibility of examining the effectiveness of Power Up in a prospective cluster randomized controlled trial and to determine the usage and acceptability of Power Up and the ability to recruit and retain participants. A feasibility trial of Power Up was conducted in specialist services and school settings. The findings from this study indicate that it is feasible to examine the effectiveness of Power Up in a prospective trial: the overall score of the Go and No Go criteria was 2 and the Trial Steering Committee have agreed on the plan to increase adherence to the criteria that were partially met. For each criterion, we have reported achievement and described facilitators and barriers to achievement to inform planning of the future trial.

The total score of the Go and No Go criteria was 2, as there were 3 criteria fully met and 2 partially met. The study will be able to proceed as the Trial Steering Committee has agreed the plan to increase adherence to the 2 amber criteria. Recruitment and retention (criteria 2 and 5) will be increased by clarifying research sites’ expectations from the outset, increasing ease of completing measures, and removing other barriers to participation (see Textbox 4). To further increase retention, barriers to downloading and using Power Up have also been removed by enabling participants to directly download the app from public app stores. As the app was still under development during the feasibility trial, Power Up was not fully available and participants were required to download Power Up using test software through the app developers; the new approach has removed this barrier. The developers have also upgraded the activity data capture system meaning we will be able to fully monitor adherence in the full trial.

The ultimate output of the project will be used by young people with emotional difficulties or other long-term conditions, aiming to empower self-management of problems. It is envisaged that Power Up will be developed for use with young people with other long-term conditions, and in other countries, however, future research is warranted to determine how Power Up should be modified for use with other conditions and in other contexts. Uptake of and engagement with novel digital technologies to support self-management of mental health difficulties and to promote decision making and well-being may be hindered if young people, their carers, teachers, and health professionals do not endorse them. The model of this study, cocreating Power Up with young people, carers, and professionals, will help to overcome this barrier by ensuring that Power Up meets the needs of these stakeholders and by providing young people with a sense of ownership in the knowledge that other young people have helped to create the resource.

**Adherence to the Go and No Go criteria.**

1. Ability to recruit and retain sites
  - *70% to 100% of sites recruited and retained: 100% (N=10/10) of sites retained (green)*.
    - Both specialist services and schools maintained high levels of engagement with the study. Clear communication about the requirements of the study from the outset was important to facilitate engagement, as was the research team attending frequent site visits, particularly in specialist services.
  - 50% to 69% of sites recruited and retained (amber).
  - 0% to 49% of sites recruited and retained (red).
2. Rates of sign up to study
  - 60% to 100% of young people signed up to participate (green).
  - *40% to 59% of young people signed up to participate: 53% (N=142/270) of participants recruited (amber)*.
    - The reported recruitment rate from the school trial was conservative as our denominators were taken from overall class lists, not the number of students present on the day. In 1 school, this particularly deflated the recruitment rate as students from eligible classes were recruited from a morning assembly that was only attended by a small proportion of students.
  - 0% to 39% of young people signed up to participate (red).
3. Rates of download and usage of Power Up
  - *60% to 100% of young people download and use Power Up: 78% (N=50/64) new users between January 2017 and February 2018 (green)*.
    - Barriers to downloading and using Power Up have been removed by enabling participants to directly download the app from public app stores (with a disclaimer that it is being used in a research trial). As the app was still under development during the feasibility trial, Power Up was not fully available and participants were required to go through a longer process to download Power Up using test software through the app developers; the new approach has removed this barrier. The developers have also upgraded the activity data capture system meaning we will be able to fully monitor adherence in the full trial.
  - 40% to 59% of young people download and use Power Up (amber).
  - 0% to 39% of young people download and use Power Up (red).
4. Young people report Power Up as acceptable in interviews
  - *70% to 100% young people report that Power Up is acceptable: 100% (N=11/11 young people) reported Power Up as acceptable in interviews (green)*.
    - All young people interviewed reported the app as acceptable. Key themes from the interviews included young people’s motivations for using the app, the impact of using the app, and barriers to use. Barriers identified related to downloading the app and technical bugs, which have been addressed by now enabling young people to download Power Up through app stores (with a disclaimer that it is being used in a research trial) and the commercial partner has resolved the technical bugs for the full deployment version of Power Up for the prospective trial. On the day of baseline assessments in 1 school, the app was not working during demonstration, likely impacting engagement and sign up (this issue has now been resolved). For participants with problems related to attention or anxiety, any technical bugs may have been of particular concern and likely to have caused frustration and disengagement.
  - 50% to 69% of young people report that Power Up is acceptable (amber).
  - 0% to 49% of young people report that Power Up is acceptable (red).
5. Completion of study measures at baseline and follow up
  - 50% to 100% of young people complete study measures at both baseline and follow up (green).
  - *20% to 49% of young people complete study measures at both baseline and follow up: 45% (N=64/142 young people) retained to follow up (amber)*.
    - In addition to the above (see criteria 2 and 3) the research team has developed Web-based versions of the baseline and follow-up questionnaires to use during the trial reducing the burden on services and schools; electronic measures were also found to be preferable with a small number of young people who completed electronic follow-up measures in the feasibility trial. The research team has also developed videos to inform young people about the study and how to use the app. The research team is confident that the aforementioned approaches will increase retention, especially in schools as students will be able to complete follow-up measures in school or at home (eg, if they are absent on the day follow-up measures are administered). One of our main strategies to increase retention will be requiring services and schools to sign a Memorandum of Understanding (MoU) agreeing to the study, which will be useful to ensure clarity about what is expected of sites and researchers. This will also help to ensure that all senior leadership and individual staff members are aware of study before it starts. In signing the MoU, services and schools will be required to elect a key contact, increasing accountability.
      In particular, research and well-being are high on schools’ agenda, meaning the full trial is likely to remain a priority to schools. As reported in the interviews, clinician engagement with, and recommendation of, Power Up are also crucial to ensure young people’s engagement (see Textbox 3). Ensuring young people, clinicians, and teachers fully understand the aims of the research, and that Power Up has been co-designed with young people, may also promote adherence to the study protocol.
    - Clinicians have a pivotal role in the adoption of technologies in health care settings, as highlighted in a recent evidence framework proposed for theorizing and evaluating nonadoption, abandonment, and challenges to the scale-up, spread, and sustainability of health and care technologies (nonadoption, abandonment, scale-up, spread, and sustainability framework) [51]. Barriers such as competing priorities, busy schedules, and engagement with the intervention are particularly salient when considering clinicians’ adoption of Power Up. Leadership and peer support for the importance of Power Up and the possible benefits for patients may help to keep Power Up a priority for clinicians. Training with a space for clinicians to work through how they would practically implement Power Up in *their* clinical practice with *their* patients may help facilitate easy integration in daily practice. Demonstrating the central role young people have played in the development of Power Up may help foster initial interest and sustained engagement over time with clinicians. Direct quotes from young people who have used it and found helpful are likely to be motivating also.
  - 0% to 19% of young people and carers complete study measures at both baseline and follow-up (red).

### Limitations

The scope of the feasibility trial was expanded to include young people from schools, in addition to young people from specialist services. Although this was based on feedback from young people, carers, and professionals that Power Up could be useful in settings beyond specialist services, it was also based on the slow recruitment rate from specialist services. We have developed plans to increase recruitment and retention for the full trial. This study was a feasibility trial, meaning definitive conclusions about the effectiveness of Power Up cannot be drawn. It was not possible to blind young people about their allocation in the feasibility trial, and it will not be possible to do so in the full trial. As with digital and psychotherapy research in general, a lack of allocation concealment and a reliance on self-report measures will be limitations that should be considered when interpreting any findings from future studies of Power Up. The Trial Steering Committee did consider using a restricted version of the app for the control condition, for example, only with the diary function. Although there is clinical equipoise, young people and clinicians indicated a strong preference not to include a *placebo* intervention, especially as young people may be unlikely to adhere to such a minimal intervention, minimizing the usefulness of comparisons. Nevertheless, in future research, independent randomization using a true randomization generator, intention-to-treat analysis, and monitoring and reporting of usage of Power Up will be methodological strengths. We will also be able to fully examine the relationship between a young person’s characteristics (eg, age, gender, and presenting problems) and the effect of Power Up.

### Conclusions

A feasibility trial of Power Up was conducted in specialist services and school settings. The findings from this study indicate that it is feasible to examine the effectiveness of Power Up in a prospective cluster randomized controlled trial: the overall score of the Go and No Go criteria was 2 and the Trial Steering Committee have agreed on the plan to increase adherence to the criteria that were partially met. This study addresses the call for feasibility trials of digital mental health interventions for young people [34,35]. Future research is needed to determine whether Power Up can be used by young people with emotional difficulties or other long-term conditions to empower them to self-manage difficulties.

## Abbreviations

CAMHS: Child and Adolescent Mental Health Service
MoU: Memorandum of Understanding
NHS: National Health Service
NIHR: National Institute for Health Research
